# A comparison of visual and auditory EEG interfaces for robot multi-stage task control

**DOI:** 10.3389/frobt.2024.1329270

**Published:** 2024-05-09

**Authors:** Kai Arulkumaran, Marina Di Vincenzo, Rousslan Fernand Julien Dossa, Shogo Akiyama, Dan Ogawa Lillrank, Motoshige Sato, Kenichi Tomeoka, Shuntaro Sasai

**Affiliations:** Araya Inc., Tokyo, Japan

**Keywords:** shared autonomy, human-robot interaction, brain-computer interface, imitation learning, multitask learning

## Abstract

Shared autonomy holds promise for assistive robotics, whereby physically-impaired people can direct robots to perform various tasks for them. However, a robot that is capable of many tasks also introduces many choices for the user, such as which object or location should be the target of interaction. In the context of non-invasive brain-computer interfaces for shared autonomy—most commonly electroencephalography-based—the two most common choices are to provide either auditory or visual stimuli to the user—each with their respective pros and cons. Using the oddball paradigm, we designed comparable auditory and visual interfaces to speak/display the choices to the user, and had users complete a multi-stage robotic manipulation task involving location and object selection. Users displayed differing competencies—and preferences—for the different interfaces, highlighting the importance of considering modalities outside of vision when constructing human-robot interfaces.

## 1 Introduction

One of the major promises of artificial intelligence (AI) and robotics is to be able automate tasks that need to be performed in the real world. Prominent use-cases for industry are warehouse robots and self-driving cars, but for individuals—particularly, physically-impaired individuals—an important domain is assistive robotics (Brose et al., 2010). Robots that can help with various household tasks would greatly improve quality of life for many people, from the elderly to those with disabilities.

For such a demographic, a dominant interaction paradigm is via brain-computer interfaces (Nicolas-Alonso and Gomez-Gil, 2012; Bi et al., 2013; Krishnan et al., 2016; Aljalal et al., 2020). Using non-invasive electroencephalography (EEG), users can control robots to accomplish tasks directly using their thoughts. However, due to the poor signal-to-noise ratio of EEG, realtime robot controller speeds, and the mental workload that would be required, direct control of a robot’s joint/end-effector space can be difficult. Although this has been a common choice (Hochberg et al., 2012; Zhu et al., 2020), hierarchical shared autonomy, whereby the user specifies a high-level task for the robot to perform, is easier and more scalable (Meng et al., 2016; Akinola et al., 2017; Lee et al., 2023). The most advanced example of such an approach is the neural signal operated intelligent robot (Lee et al., 2023), which combines the steady state visually evoked potentials and motor imagery EEG paradigms to allow a user to select an object from a tabletop, and where and how to interact with it.

With advances in “end-to-end” AI (LeCun et al., 2015), the landscape of robot control has gradually also shifted to shorter pipelines and components that are learned. In particular, advances in natural language processing and computer vision (Radford et al., 2021) have spurred language-conditioned imitation learning for robotics (Ahn et al., 2022; Shridhar et al., 2022a; Brohan et al., 2022; Shridhar et al., 2022b; Driess et al., 2023; Goyal et al., 2023). These range from sample-efficient solutions relying on hybrid AI techniques (Shridhar et al., 2022a; Shridhar et al., 2022b; Goyal et al., 2023) to large-scale models (Ahn et al., 2022; Brohan et al., 2022; Driess et al., 2023), but both allow robots to learn how to perform a range of tasks using a single algorithm, and with human-interpretable natural language commands. We posit that such methods are therefore promising for human-robot interaction, and hence BCI with robot systems should be designed using these.

Considering such robot systems would be capable of performing a range of tasks, a question that arises is how best to direct them. Furthermore, would a visual interface, that is the most common choice in these scenarios, be best, or would an auditory interface, which allows the user to focus their visual attention on the robot, be preferred? In this work we investigate this question by designing and testing novel context-dependent visual and auditory BCIs based on the commonly-used oddball paradigm (Squires et al., 1975). We then perform a user study, using the language-conditioned Perceiver-Actor (Shridhar et al., 2022b) model to control a robot arm to perform a multi-stage task, cleaning up a table. We find that users prefer different interfaces, and hence personalised BCIs (Ma et al., 2022) should be considered when developing future BCI + robot systems.

While there have been several studies comparing visual and auditory stimuli under the P300 paradigm (Subsubsection 2.2.1; Furdea et al., 2009; Belitski et al., 2011; Käthner et al., 2013; Oralhan, 2019), no comparative studies exist during the task of operating a robot. In contrast to these studies, which found higher performance associated with the visual interface, our results (Section 3) indicate that different users can have different affinities for sensory modalities when directing a robot manipulation task. Furthermore, whilst NOIR presents an elegant BCI-robot pipeline with multitask policies and robot learning, their use of predefined parameterised primitive skills is less scalable than our use of language-conditioned imitation learning, which we believe is a first in the context of BCI-robot setups.

## 2 Materials and methods

### 2.1 Task

**FIGURE 1:**
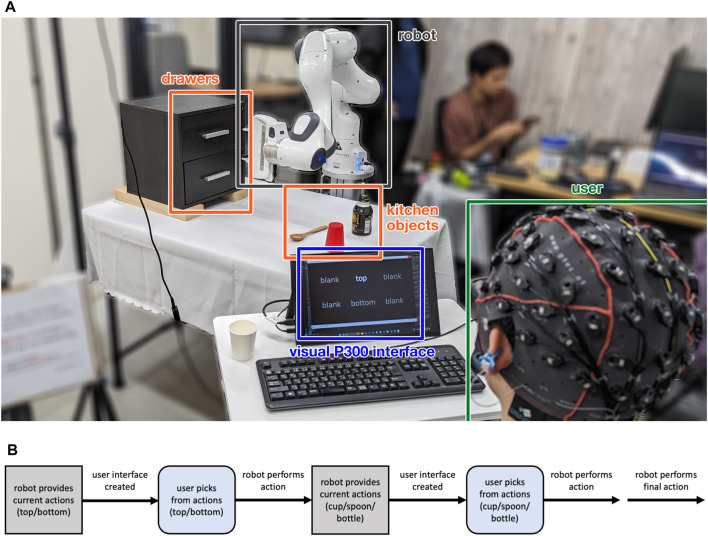
**(A)** Experimental layout. The user with the EEG cap sits in front of a small table facing the robot, with a display (for the visual P300 interface only). The robot is behind a table with chest of drawers and three kitchen objects: a cup, a spoon, and a bottle. **(B)** Multi-stage task control flow. After the robot environment is initialised, the robot provides a set of actions that can be performed. These actions are used to create the user interface. After an action has been decoded by the user interface, the robot performs the chosen action, and then provides the next set of actions. This generic process can be repeated; however, in our particular task there are two decision points for the user (pick a drawer to open, and item to pick and place), after which the robot performs a third and final action (close the drawer) autonomously.

We designed a simple, multi-stage task to test the efficacy of the two different user interfaces under a shared autonomy paradigm. The user is tasked in directing a robot arm to tidy up a mock kitchen table (Figure 1A). Firstly, the robot must open either the top or bottom drawer of a chest of drawers, and secondly, pick up either a cup, spoon, or bottle, to put into the open drawer. Once a full task is completed, the robot arm closes the open drawer, and another instantiation of the task is provided to the user, e.g., open the bottom drawer and pick up the bottle. With our multi-task imitation learning setup, described in detail in Section 2.5, we can train the robot to perform different manipulation tasks in a scalable manner.

The user is provided with decisions at two points: firstly, to choose between the two drawer locations, and secondly, once the robot arm has opened the drawer, to choose between the three kitchen objects. The interface gives the user the task specification at the beginning of each trial, and after task completion, the environment is manually reset (the object is put back on the table) before the next trial begins. The control flow for a single, multi-stage task is shown in Figure 1B.

For the user, we measure subtask success, i.e., decoding the correct object to place in the wrong drawer results in 1 success (out of a maximum of 2) for the trial. For the robot, we also measure subtask success, including closing the drawer. As an example, opening the decoded drawer and picking the decoded item, but failing to close the drawer results in 2 successes (out of a maximum of 3) for the trial. We consider user and robot success independently. If the user selects the wrong object, but the robot picks up the object that was selected, then it is a failure for the user, but a success for the robot. If the robot fails at its action at any stage, a human experimenter completes the action in order for the experiment to continue.

### 2.2 User interfaces

BCIs can be categorised into 3 different interaction types: active, reactive, and passive (Hramov et al., 2021). Active BCIs are effortful, and require the user to make use of some form of mental imagery (most commonly motor imagery) to control a system, whilst passive BCIs detect the user’s mental condition (e.g., attention or emotional state) to provide feedback to an external system. Reactive BCIs generally provide the best signal-to-noise ratio, as they rely on the user’s brain reacting to external stimuli, and hence we chose to design our interface based on this interaction type.

#### 2.2.1 Oddball paradigm

Our interface relies on detecting the event-related potential (Luck, 2012) associated with oddball stimuli (Squires et al., 1975); this is the response of the brain to the rare occurrence of a target stimulus amongst sequences of non-target stimuli. When combined with decision making, the oddball paradigm elicits the P300 response (Polich, 2007), which is a higher amplitude signal occurring at the parietal lobe after 300 ms beyond the presentation of the target stimulus.

Various experimental conditions affect the P300 elicited: stimulus categorisation time increases latency (Kutas et al., 1977), greater similarity between target and non-target stimuli reduces the difference in magnitude between their responses (Azizian et al., 2006), and the magnitude of the response increases with target stimuli rarity (Donchin and Isreal, 1980). In short, the target stimulus must be relatively surprising (Donchin, 1981). Typical stimuli include coloured circles for visual interfaces and simple tones/sounds for auditory interfaces (Fayaz et al., 2020).

However, without a natural mapping (Norman, 2013) from interface controls to their actions in the real world, users can find an interface unintuitive. Therefore, we made a decision to trade off stimuli simplicity for a more flexible, potentially open-ended design.

We designed our interface based on the well-studied P300 speller interface (Farwell and Donchin, 1988). The standard P300 speller consists of a grid of characters that flash in a random sequence, with the user eliciting a P300 response when the target character flashes. Instead, our task presents either two (“top” or “bottom”) or three (“cup”, “spoon” or “bottle”) semantic choices at each decision point. As iterating between few options makes all stimuli (target and non-target) presentations surprising, we added “blank” items to our interface (Figure 1A), to make a total of 6 items to choose between; this was a tradeoff between increasing the target rarity and the speed with which all items could be iterated over. To the best of our knowledge, research based on P300 spellers have only considered a minimum of 4 items, and so the use of blank/null items is a novel solution to using the tried-and-tested P300 speller paradigm with a small number of choice items.

#### 2.2.2 Visual user interface

Our visual interface is based on the standard P300 speller interface, with a 2D matrix of monochrome items arranged on a plain background (Farwell and Donchin, 1988). When the robot requires a decision from the human, it sends a list of the choices to the interface, which then makes and displays the matrix of items (Figure 1A). The 6 items (including blanks) are laid out in a 2 × 3 grid with a black background. The current stimuli text flashes white, and otherwise all text is grey.

The advantages of the visual user interface are: humans have a stronger ERP response to visual stimuli (in contrast to auditory stimuli (Fayaz et al., 2020)), there is a larger design space for visual interfaces, they can have a high information density, and in general there is more research on visual BCI (Gao et al., 2014). The major disadvantage is that the user has to focus away from the environment and on the interface, resulting in divided attention (Liu, 2001).

#### 2.2.3 Auditory user interface

There have also been several attempts to build auditory analogues of the P300 speller, typically mapping simple audio stimuli to the rows and columns of the visual version (Furdea et al., 2009; Klobassa et al., 2009). We instead chose to directly play back the choices through speech in a random sequence, generated by a pretrained SpeechT5 text-to-speech model (Ao et al., 2022).

With the visual interface, all items are presented simultaneously, with a maximum of one item being highlighted at once. However, this is not feasible with the auditory interface, which uses words spoken sequentially. The onset of speech can be surprising, therefore a constant stream of white noise was added in the background to mitigate this effect; the white noise is played continuously, and the words are spoken at regular intervals. We believe this does not have a significantly negative effect on ERP detection, as prior research has shown that P300 responses can still be elicited in the presence of background white noise (Salisbury et al., 2002).

In contrast to the visual user interface, there is less flexibility in designing the auditory interface, but it does allow the user to maintain their visual focus on the scene in front of them. As there is less indirection, the auditory interface can induce greater feelings of having “mind control” over the robot, increasing engagement.

#### 2.2.4 Experimental schema

In our user study (Section 2.6), we first collect EEG data using just the interface to train decoders for each user, and then perform online decoding with a real robot. The main difference in the interface between these two phases is that with online decoding, the robot performs the action once chosen.

For each trial (requiring first a choice of location, and then a choice of item), each choice proceeds as follows for the visual interface. Firstly, the target is displayed for 5 s. Then a neutral interface (all items grey) is displayed for 0.6 s. Then the interface loops through a random permutation of the items (all words are flashed once in one “loop”), with a stimuli presentation time of 0.25 s and a neutral inter-stimuli display time of 0.05 s, with 5 loops for data collection and up to 10 loops for online decoding. We record the EEG data associated with the stimuli presentation and inter-stimuli display times as a single EEG epoch. During data collection we collect data for all choices that may occur when interacting with the environment (“top” and “bottom”, and separately “cup”, “spoon” and “bottle”).

For the auditory interface, we had to increase the stimuli presentation time to 0.5 s to avoid words being cut off, increased the delay between stimuli presentations to 0.55 s to ensure smoother speech playback, and also introduced a ping as a cue between the specification of the target and the loop to reduce surprise.

### 2.3 EEG

We acquired EEG data with a 64-channel g. SCARABEO g. GAMMAcap2^1^ with active electrodes at positions specified by the extended international 10–20 system. All 64 electrodes were used for decoding. AFz was used as the ground electrode, with both earlobes as reference. Conductive gel was applied to each electrode until the impedance was under 20 k Ω. The data stream was collected at 512Hz, with a notch filter at 50 Hz to reduce line noise, and a bandpass filter at 0.1–30 Hz, which is standard for ERP detection (Luck, 2012). Furthermore, we automatically rejected any epochs which had a maximum peak-to-peak signal amplitude of over 5000 μV using MNE’s drop_bad ^2^ method; this value was chosen conservatively, allowing many epochs with artefacts to be included during recording/decoding.

### 2.4 Decoder

For each user and interface, we trained an ensemble of 10 support vector machine (SVM) classifiers on the log spectrogram (power spectral density of the short-time Fourier transform) of the signal. The spectrogram was calculated using scipy’s spectrogram ^3^ function with segments of size 32, resulting in 7,616 features per data point. The SVMs were trained using stratified *k*-fold cross-validation, which means that each SVM was trained on a different 90% split of the data. To combat the class imbalance with ERP data, we weighted samples proportionally to the inverse of their class frequencies. Other than the weighted loss, all SVMs were trained using the default settings from the scikit-learn library (Pedregosa et al., 2011). During decoding, we used majority voting over the ensemble of SVMs to detect ERPs; in the event of a tie (5/10 predictions of an ERP), then we count it as a detection. If a maximum of 10 presentations of all stimuli passes without an ERP being predicted, we consider it a timeout (decoder failure) and proceed to the next subtask.

### 2.5 Robot

For our experiments we used a 7 DoF Franka Panda robot arm, placed in front of a mock kitchen table (Figure 1A). For colour and depth input, we also placed an Intel RealSense D435i camera opposite the robot.

To control the robot, we used PerAct (Shridhar et al., 2022b), a language-conditioned, multitask imitation learning algorithm. PerAct takes in a voxel view of the scene, proprioceptive inputs (gripper open/close status and position), and a natural language instruction (using a pretrained CLIP language encoder; Radford et al., 2021). These are then processed by a Perceiver IO Transformer (Jaegle et al., 2022). Finally, the output of the Transformer is decoded to produce the desired gripper position (specified in voxel space; James and Davison, 2022), rotation, open/close status, and motion planning mode. Instead of directly outputting actions for the robot to execute, PerAct uses a traditional motion planner—by default, RRT-Connect (Kuffner and LaValle, 2000) within the ROS MoveIt! package (Chitta et al., 2012). PerAct is trained using supervised learning on input-action tuples from a set of demos; we trained a single model on 7 tasks with the corresponding language commands: “open the top/bottom drawer”, “pick and place the cup/bottle/spoon”, and “close the top/bottom drawer”. We collected 60 demos with teleoperation (10 for opening the drawers +20 for closing the drawers +30 for picking up and placing the items in the drawer), over a span of 6 h, to train our multitask PerAct model. We randomised the initial positions of the small objects during training and testing, and adjusted the drawer position and orientation slightly while maintaining its position at the right side of the table. Our changes against the default PerAct settings include restricting the input and position output to a 60^3^ voxel grid (as opposed to the default of 100^3^) due to limited GPU memory, and using keypoints from our teleoperation setup (as opposed to calculating them heuristically).

### 2.6 User study

We recruited a total of 7 volunteers for our study (1 female, average age of 29 years, with a standard deviation of 2.07 years). Users were initially given a briefing on the purpose of the study and experimental protocol, and if they consented, proceeded to the actual experiment. Our study was given ethical approval by the Shiba Palace Clinic Ethics Review Committee.

After setting up a user with the EEG cap, we presented them with one of the user interfaces and a series of randomised targets in order to collect training data to calibrate the user-specific EEG decoder. We collected approximately 20 min of calibration data per user, per interface, which consisted of 5 presentations of each of the 5 targets (top/bottom/cup/spoon/bottle), alongside non-target presentations (with each loop of the interface having 1 target item +5 non-target items), 10× for the visual interface, and 5× for the auditory interface. This resulted in a maximum of 1,500 and 750 epochs of data per user (given that some epochs are automatically rejected), for the visual and auditory interfaces, respectively. This data was used to train an ensemble of decoders (Subsection 2.4).

During the online decoding experiments, we measured decoding and robot success independently (Subsection 2.1). If the decoder predicted an ERP on a blank stimulus, or timed out, we sent a random object to the robot to interact with. Online decoding consisted of a total of 12 trials (2× all combinations of choices). After the online decoding trails, the user was given a questionnaire with 18 7-level Likert items.

We then repeated data collection and online decoding with the other interface. To control for learning effects in the results, we split users so that half used the visual interface first, and the other half used the auditory interface first. In total, the entire procedure took up to 3 h per user.

## 3 Results

### 3.1 Decoder

**FIGURE 2:**
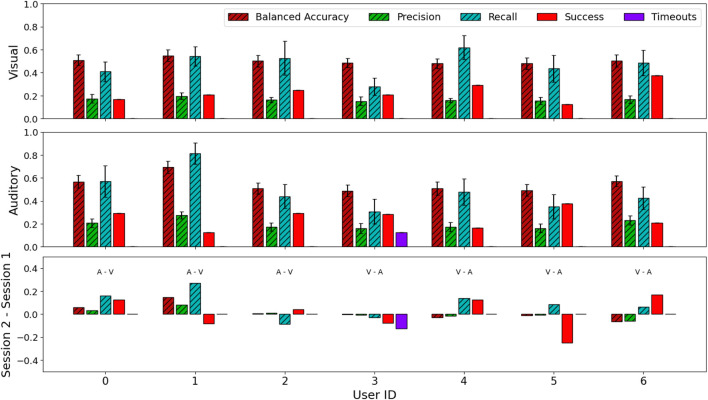
EEG decoding results: hatched bars correspond to decoder training, and plain bars correspond to online decoding. The average balanced accuracy, precision and accuracy across users was 0.50 ± 0.05, 0.17 ± 0.03 and 0.47 ± 0.15 for the visual interface, and 0.55 ± 0.09, 0.20 ± 0.06 and 0.48 ± 0.19 for the auditory interface, respectively. Average success was 0.23 ± 0.42 for the visual interface, and 0.25 ± 0.43 for the auditory interface. Only one user experienced timeouts (3, on the auditory interface).

Our primary results are based on 336 online decoding choices (7 users × 2 interfaces × 12 trials × 2 choices). On average, during online decoding, users had a success rate (rate of successfully selecting the target item) of 0.23 ± 0.42 for the visual interface, and 0.25 ± 0.43 for the auditory interface, with only one user experiencing timeouts. Firstly, we note that our success rates are a lower bound on the success of a real deployed system, as, in this study, we allow the interfaces to decode ERPs on the “blank” items, whereas in practice we could restrict the interface to only select from valid items. Secondly, different users had different proficiencies, with some being notably more successful with one interface over the other (Figure 2). This supports the idea of personalised BCIs (Ma et al., 2022), as different users may prefer to interact using completely different modalities. Indeed, although most users tended to find the auditory interface harder to use, some preferred it, as they could directly observe the robot instead of looking at a separate screen.

There was little difference on the decoder training metrics between interfaces, with a balanced accuracy on *k*-fold cross-validation of 0.50 ± 0.05 for the visual interface, and 0.55 ± 0.09 for the auditory interface. Precision and recall was 0.17 ± 0.03 and 0.47 ± 0.15 for the visual interface, and 0.20 ± 0.06 and 0.48 ± 0.19 for the auditory interface. *A priori*, we believed that precision would be the most important metric, as minimising the amount of false positives would reduce the chances of the decoder selecting the wrong object. However, when calculating the Pearson correlation coefficient (Freedman, 2009) between all three decoder training metrics and online success, we did not find any strong correlations. We believe that this is due to the domain shift between training and online decoding, as we discuss below.

As users interacted with the two interfaces sequentially, we anticipated that there may be learning effects. However, when we compare the difference in performance between the second and first sessions (Figure 2), only half the users experienced an increase in the success rate, regardless of whether they used the auditory or visual interfaces first. Interestingly, the difference in success rate is more pronounced than in the balanced accuracy, which we believe is due to the domain shift between collecting data from the interface without interacting with the robot, and online decoding where the user is engaged with trying to control the robot. Qualitatively, most users felt pressure during the online decoding sessions, and users who were able to relax the most achieved higher success. As attention and time-pressure can affect P300 responses (Hohnsbein et al., 1995), the decoders would need to extrapolate to out-of-distribution data. Although data collection did not involve robot interaction to speed up the process, finding a way to make the offline and online decoder processes more similar should not only improve online success, but hopefully make the data collection more engaging for users.

**FIGURE 3:**
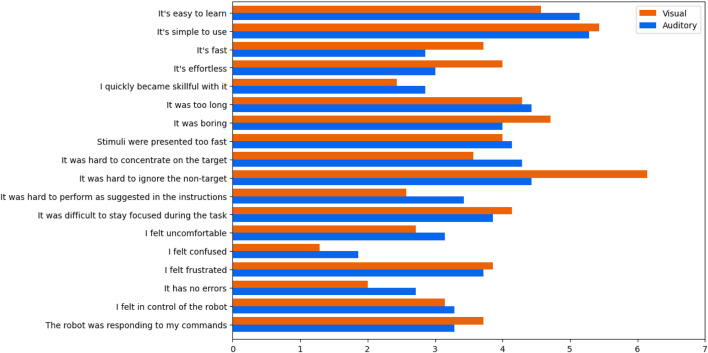
Usability questionnaire results. Users understood the interfaces without much difficulty, but were frustrated with poor decoding accuracy. The most significant difference between the interfaces was the ability to ignore the non-targets, with users finding this particularly difficult with the visual interface.

The questionnaire results were mixed (Figure 3). On the positive side, users seemed to understand the task and interface well. On the negative side, users were not so engaged, and were aware that decoding had a lot of errors. Feedback on the two interfaces only differed by more than 1 point on 2 items: the visual interface required less effort, and it was easier to ignore the non-target with the auditory interface. The latter observation is supported by a *post hoc* analysis of the training data, as P300 components were found in the non-target epochs of the visual interface. Redesigning the interfaces to reduce interference from non-target stimuli would greatly improve usability and decoder performance.

### 3.2 Robot

**FIGURE 4:**
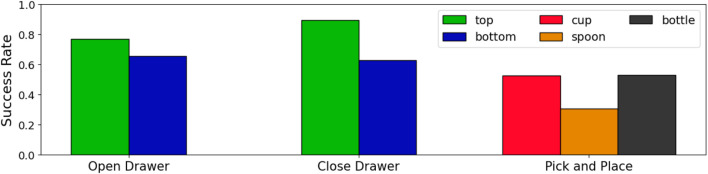
PerAct success rates. On average, PerAct had a success rate of 72% on opening the drawer, 78% on closing the drawer, and 45% on picking and placing objects.

Overall, PerAct was 65% successful, over a total of 504 subtasks (7 users × 2 interfaces × 12 trials × 3 subtasks). The success was highly (sub)task-dependent (Figure 4), with closing the top drawer being easiest (90% success), and picking up the spoon hardest (31% success). One of the main difficulties for PerAct with picking up objects is predicting the gripper position precisely enough to prevent slippage—an issue we believe could be ameliorated with a higher resolution voxel grid and better point cloud noise filtering. A few failures came from picking up the wrong object, which could have been better handled by adding segmentation-conditioning to PerAct (Akiyama et al., 2023). However, the latter algorithm had only been tested on different object types, and not on referring object detection, e.g., detecting the “top drawer”. Unfortunately, open-set object detection algorithms that can perform referring object detection (Liu et al., 2023) still have poor top-1 accuracy on images from our robot environment. Finally, some of our failures came from RRT-Connect planning paths that came too close to obstacles, triggering the robot’s automatic safety mechanism. Robot learning methods such as PerAct that predict keypoints can accomplish more tasks, with greater safety, by instead using an ensemble of path generation methods (James and Abbeel, 2022), and so we hope to integrate these in the future.

## 4 Discussion

In this work we focused on what we see as the upcoming setting for BCIs with robots—multi-task (imitation) learning controllers, combined with context-dependent interfaces. We trained a PerAct agent (Shridhar et al., 2022b) to open and close drawers, and pick up and place small household objects into open drawers, enabling a user to direct a real robot arm to tidy up a table. In order to control the robot, we designed novel user BCIs, trialling both visual and auditory modalities. Users displayed preferences for different interfaces, highlighting the importance of personalisation in BCI design (Ma et al., 2022).

The main limitation of our study is the low success rate of the online decoder. Although all users were able to achieve above chance success (1/6, the reciprocal of the number of items presented in one loop of the interface) with at least one modality, none were able to achieve significantly higher than this. One issue was the limited training data—we sacrificed potential performance for improving the users’ comfort. We inferred from user feedback that the increase in mental workload when operating the real robot could also have reduced the performance of the decoder, as it is known that the amplitude of the P300 signal is smaller when mental workload is high (Gopher and Donchin, 1986; Kramer et al., 1986; Wintink et al., 2001).

One way to improve the performance of our EEG decoder would be to collect more training data, or use transfer learning methods (Wan et al., 2021). More training data would also enable us to use more sophisticated models for classifying EEG signals (Lawhern et al., 2018). Similarly, we expect that PerAct’s success rates could be improved by collecting more demos. Finally, we would like to highlight that the recent NOIR system (Lee et al., 2023) presented several innovative design choices for BMI-robot pipelines that are orthogonal to our work, and we believe combining these with elements of our approach could lead to even more flexible and scalable BMI-robot setups in the future.

## Data Availability

The datasets presented in this article are not readily available because of privacy reasons. Requests to access the datasets should be directed to KA, kai_arulkumaran@araya.org.

## Footnotes

1
https://www.gtec.at/product/g-scarabeo-eeg-electrodes/

2
https://mne.tools/stable/generated/mne.Epochs.html#mne.Epochs.drop_bad

3
https://docs.scipy.org/doc/scipy/reference/generated/scipy.signal.spectrogram.html
